# Vestibular impact of Friedreich ataxia in early onset patients

**DOI:** 10.1186/s40673-020-00115-z

**Published:** 2020-05-28

**Authors:** A. Maudoux, N. Teissier, M. Francois, Th. Van Den Abbeele, C. Alberti, I. Husson, S. R. Wiener-Vacher

**Affiliations:** 1 Center for Balance Evaluation in Children (EFEE), Otolaryngology Department, Assistance Publique des Hôpitaux de Paris, Universitary Robert-Debré Hospital, F-75019 Paris, France; 2 Université de Paris, U1141, NeuroDiderot, Inserm, F-75019 Paris, France; 3 Clinical Epidemiology unit, Assistance Publique des Hôpitaux de Paris, Robert-Debré Hospital, F-75019 Paris, France; 4 Unité INSERM CIC-EC 1426, UMR-S 1123, F-75019 Paris, France; 5 Functional Rehabiliation Unit, Assistance Publique des Hôpitaux de Paris, Robert-Debré Hospital, F-75019 Paris, France

**Keywords:** Friedreich Ataxia, Hearing, Vestibular function, Auditory neuropathy

## Abstract

**Background:**

Friedreich ataxia (FRDA) is the most frequent form of inherited ataxias. Vestibular and auditory assessments are not commonly part of the check up for these patients despite hearing and balance complaints. Screening of vestibular and auditory function was performed in a large group of young patients with genetically confirmed FRDA.

**Methods:**

Our study included 43 patients (7–24 years of age). A complete vestibular assessment was performed including the canals function evaluation at 3 head velocities (bithermal caloric test, earth vertical axis rotation (EVAR) and head impulse test (HIT)) and otolith function evaluation (cervical vestibular evoked myogenic potentials). Information regarding the hearing evaluation of the patients were also retrieved including impedance tympanometry, distortion product otoacoustic emissions (DPOAEs), air and bone conduction audiometry and auditory brainstem response (ABR).

**Results:**

Vestibular responses were impaired for canal responses (only at high and middle head velocities) and vestibulospinal otolithic responses. Abnormal neural conduction in the central auditory pathways was frequently observed. Oculomotor abnormalities were frequent, mostly hypermetric saccades and gaze instability. Inhibition of the vestibulo-ocular reflex by fixation was normal.

**Conclusions:**

We show that Friedreich ataxia, even at onset, frequently associate saccadic intrusions, abnormal ABRs and decreased vestibulo-ocular and vestibulospinal responses progressing over time. These sensory impairments combined with ataxia further impair patient’s autonomy. These vestibular, auditory and visual impairments could be used as markers of the severity and progression of the disease. Adding vestibular and auditory testing to Friedreich patient’s evaluation may help physicians improve patient’s management.

## Background

Friedreich ataxia (FRDA) [1] is the most frequent form of inherited ataxias with an estimated carrier frequency of 1/50–1/100 in the general population [2] and a disease incidence between 1/20000 and 1/250000 [3]. FRDA is an autosomal recessive degenerative disease frequently starting around puberty. It is characterized by a progressive gait ataxia, limb weakness, apparition of Babinsky sign, loss of deep tendon reflex, dysarthria, and later skeletal deformities [4, 5]. In addition, hypertrophic cardiomyopathy is found in almost all patients [6], diabetes mellitus in about 12% and carbohydrate intolerance in 49% [7]. The natural history of FRDA is a slow progression of the symptoms over decades with increasing dependence for daily activities. Today, no treatment is available to delay the progressive course of the disease.

FRDA is due to a mutation of the FXN gene on chromosome 9. In most patients the anomaly is an unstable GAA triplet repeat expansion within intron 1 of FXN [8]. Where normal chromosomes contain 7–22 units, FRDA chromosomes can carry 100 to 1300 GAA repeats in both copies of the FXN gene. The latter codes for frataxin, a protein that is required for mitochondria to function properly. A deficiency in frataxin leads to progressive central and peripheral nervous system damage. Phenotypic variability of the disease correlates with the size of expanded GAA repeats. Shorter expansions are often associated with late onset of the disease, slower progression and absence or mild cardiomyopathy and diabetes [9].

Previous studies on FRDA patients report abnormal vestibular functions. These studies suggest that the vestibular system can be impaired for all head velocities since abnormal vestibulo-ocular reflexes (VOR) were reported for caloric test, rotatory tests and head impulse test (HIT) [10–13]. However, little is known concerning the peripheral or central origin of the vestibular deficit. The aim of our study was to perform a complete vestibular evaluation associating both canal and otolithic explorations in order to better localize the neurophysiological impairment. In addition to thorough vestibular testing, information regarding the auditory and oculomotor functions were retrieved.

Hearing tends to be normal in FRDA patients, and most studies show normal or near to normal audiometric thresholds [4, 14–16]. However, these patients frequently report speech perception problems [15, 17]. Some studies reported a high prevalence of either absent or distorted 8th nerve and auditory brainstem electrophysiological responses in FRDA [10, 18–21].

Oculomotor abnormalities are common in FRDA and typically include saccadic dysmetria, impaired smooth pursuit, gaze instability and gaze nystagmus with ocular flutter or square wave jerks (SWJ) [10, 11, 22]. It is so frequent that the presence of such signs, like ocular flutter or square wave jerks, in subjects with progressive ataxia is strongly suggestive of FRDA [11]. Furthermore, the presence of abnormal eye movement at early onset of FRDA is a sign of disease severity [11, 22].

## Materials and methods

### Subjects

Forty-three subjects with genetically characterized FRDA (18 boys and 25 girls) aged 7 to 24 years were included in the study. The investigation adhered to the principles of the Declaration of Helsinki and was approved by our Institutional Human Experimentation Committee (Comité de Protection des Personnes CPP Ile de France; P070123 AOM07049; EudraCT 2008–003360-19). Informed consent to participate in the study was obtained from the patients and/or their legal representative. For most subjects (*n* = 40/43) the vestibular, hearing and oculomotor evaluations were performed as part of a study on the effect of Pioglitazone on FRDA. These subjects underwent two evaluations, one at the inclusion and one after the 2 years follow up (Effect of Pioglitazone Administered to Patients with Friedreich’s Ataxia: Proof of Concept (ACTFRIE), ClinicalTrials.gov identifier: NCT00811681). For these 40 children and young adults, only the results of the first initial evaluation were used in our study. The 3 other subjects, not included in the ACTFRIE study, were addressed to our department as part of a multidisciplinary clinical evaluation protocol for diagnosed FRDA children. The number of GAA repeats in both copies, age at onset and duration of the disease were recorded only for patients participating in the ACTRFRIE protocol. Disease severity was assessed using clinical scales: the International Cooperative Ataxia Rating Scale (ICARS) [23], the Friedreich Ataxia Rating Scale (FARS) [24] and the Scale for Assessment and Rating of Ataxia (SARA) [25].

The ICARS is a rating scale assessing the impact of ataxia using four clinically subscales scored separately: posture and gait disturbances (PG), kinetic functions (KF), speech disorders (SD), oculomotor disorders (OD). Scores for each subscale quantify the impact of ataxia in each of these clinically important areas. The sum of the subscales score gives a total score ranging from 0 to 100 [26]. The FARS is scored out of 159 and include three subscales evaluating ataxia, performance on daily living activities and neurological symptoms [27].. The SARA scale assesses a range of different impairments in cerebellar ataxia. It is made up of 8 items related to gait, stance, sitting, speech, finger-chase test, nose-finger test, fast alternating movements and heel-shin test for a total score of maximum 40 [25]. For all three clinical scales, a higher score indicates greater level of functional disability.

### Vestibular and neurologic evaluation

The vestibular system responds to sensory inputs across a range of frequencies going from low frequencies of approximately 0.05 Hz (e.g. postural sway) to high frequencies around 5 Hz during rapid head movements [28, 29]. Canal function was evaluated at different velocities with 1- bithermal caloric test (33 °C and 44 °C) for low velocities, 2- earth vertical axis rotation (EVAR) with 40°/s^2^ acceleration and deceleration along a vertical axis for medium velocities [30] and 3- head impulse test (HIT) to evaluate the 6 semicircular canals at high velocities [30]. Otolithic function was assessed by using cervical vestibular evoked myogenic potential (c-VEMP) with short tone bursts (750 Hz, 4.1/s and 6-ms duration) delivered by air and bone conduction with a control of the electromyogram level for each stimulation (Difra-Neurosoft® system Bruxelles, Belgium) [31]. This later characteristic allows the selection of the responses obtained for the same level of EMG. The EVAR test was performed using a computer-driven rotatory chair (SAMO®, Caen, France), and the vestibulo-ocular responses (VORs) were recorded by electronystagmography more adapted to young children than video recordings. Description of the EVAR experiment apparatus and protocol has been described in detail in previous publications [31].

For the bithermal caloric test, the Jongkees formula was applied [32]. Values for relative valence and directional preponderance for children were considered normal when < 15%. The responses to bithermal caloric test were categorized as either normal, absent bilaterally, partially and symmetrically impaired (bilateral symmetric hyporeflexia), or partially asymmetrically impaired (one side being either areflexic or hyporeflexic compared to the other side).

The HIT test was either normal (no catch-up saccade observed) or abnormal (presence of a catch-up saccade in at least one direction). Visible catch-up saccades indicate that the canal has lost at least 75% of its function.

For the c-VEMP, we studied the P and N latencies (ms), amplitude of P-N (mV) and the response thresholds (dB). The VEMP results could either be normal (present on both sides with a symmetric P-N amplitude at 100 dBHL), absent bilaterally (with no detectable P and N at 120 dBHL), partially symmetric (positive responses with thresholds > 100 dBHL), or partially asymmetric (difference of thresholds between sides > 10 dBHL and/or P-N amplitude difference exceeding 100 μV between the 2 ears).

For the EVAR test, we measured the time constant and maximal initial slow phase velocity of the VOR. Canal VOR time constant was calculated from the curve of the decay of slow phase velocities over time (measured from the start of the fastest slow phase until the extinction of the nystagmus); it corresponded to the time necessary to cover about one-third of the total area subtended by the slow phase velocity/time curve [31].

### Hearing evaluation

A complete hearing assessment was performed including impedance tympanometry, distortion product otoacoustic emissions (DPOAEs), separate air and bone conduction audiometry and auditory brainstem response (ABR). Impedance audiometry was categorized as follows: type A, normal; type B, flat; type C, shifted negatively and type Ad, steeper maximum rising above the normal region (deep curve). DPOAEs were collected in the external ear canal in response to pairs of equilevel pure tones at frequencies f1 and f2 (f2/f1 = 1.20; L1 = L2 = 65, 70 and 75 dB SPL), with f2 swept from 0.5 to 8 kHz in ¼ octave steps (Ero Scan Maico). The DPOAEs could either be normal (signal to noise ratio > 15 dB), present but low in amplitude (signal to noise ration above 7 dB but lower than 15 dB) or absent [33]. Audiometry was performed with a Madsen Conera Audiometer using TDH39 Headset and B-71 Bone vibrator. The average of pure-tone auditory thresholds at 0.5, 1, 2 and 4 kHz served as index of hearing sensitivity. The average pure-tone auditory threshold using air conducted stimuli was categorized as follows: normal, < 20 dBHL; mild sensorineural hearing loss (SNHL), 21 to 40 dBHL; moderate SNHL, 41 to 70 dBHL; severe SNHL, 71 to 90 dBHL; and profound SNHL, > 90 dBHL (audiometric classification of hearing impairments, recommendation from the International Bureau of Audiophonology – BIAP). In case of abnormal air conducted auditory threshold, bone conducted auditory threshold was performed looking for conductive hearing loss.

For ABR recordings, we used the OtoAccess equipment from Interacoustics® (v1.2.1). ABRs were collected between two scalp electrodes placed on the forehead and ipsilateral mastoid and averaged in response to repeated clicks (80 dB nHL, alternate polarity, presentation rate 28.7/s, delivered by TDH-39P headset, four consecutive trials of 250 clicks). The ABRs were either normal, showed increased latencies, progressive dys-synchronization (normal ABRs in the first trial but progressive increased latencies and dys-synchronization in consecutive trials) or immediate dys-synchronization (present at the beginning of the stimulations).

### Oculomotricity study

The oculomotricity assessment included the evaluation of the gaze stability, the ocular saccades, the ocular pursuit and the optokinetic nystagmus (OKN). Saccades were recorded with electro-occulography [34]. The target included nine LED 19 cm apart presented on a vertical board. The board was placed at 1 m from the patient’s eyes who was sitting in a chair and the LED were randomly lightened in location and time without overlap. Velocities gain and precision of the saccades were analyzed.

### Statistical analysis

Statistical analysis was undertaken using R software v3.3.3 [35]. For numerical values, difference between groups were compared with nonparametric tests (U Mann-Whitney and Kurskall-Wallis) since normality could not be assumed (small sample size of several subgroups *n* < 15). For categorical variables, Chi-square or Fisher’s exact test were used to test for independence and compare proportion. Correlation analyses were performed using Pearson’s correlation after controlling for normality.

## Results

### Population characteristics

The 43 subjects included in the study had a mean age of 16.8 years (S.D. 4.6, range 7.67–24.97) at time of evaluation. The mean duration of the disease was 90.7 months (SD 43.8, range 12.55–204.1). The mean age at onset was 9.4 years old (SD 4, range 2–16.43). The genetic analysis was retrieved for the 40 subjects included in the ACTFRIE protocol (for average number of triplets repeats); a mean 702 triplet repetitions was identified on the first allele (SD 145, range 400–966) and a mean 913 triplet repetition on the second allele (SD 186, range 500–1366). Forty subjects performed the ICARS scale evaluation. The mean ICARS total score of our population was 34.9 (SD 13.7, range 5–70). Regarding the four subscales, the mean posture and gait disturbances score was 14.8 (SD 6.7), the mean kinetic functions score was 17.4 (SD 7.7), the mean speech disorders score was 1.3 (SD 1.2) and the mean oculomotor disorders score was 1.4 (SD 1). Thirty-eight subjects completed the FARS scale. The mean FARS score was 7.2 (SD 17.4, range 18–102.5). Forty subjects completed the SARA scale. The mean SARA score was 14.8 (SD 5.9, range 1.5–30.5). All these data are presented in Table 1.

**Table 1:** Population’s characteristics

	Mean ± SD	Range
**Age at visit (years)**	16.8 ± 4.6	7–24
**Age at disease onset (years)**	9.4 ± 4	2–16
**Disease duration (months)**	90.7 ± 43.8	12–204
**Longer GAA repeat**	913.1 ± 186.3	500–1366
**Shorter GAA repeat**	702.3 ± 145.5	400–966
**ICARS**	34.9 ± 13.7	5–70
**FARS**	67.2 ± 17.4	18–102
**SARA**	14.8 ± 5.9	1.5–30.5

### Vestibular function assessment

**Fig. 1:**
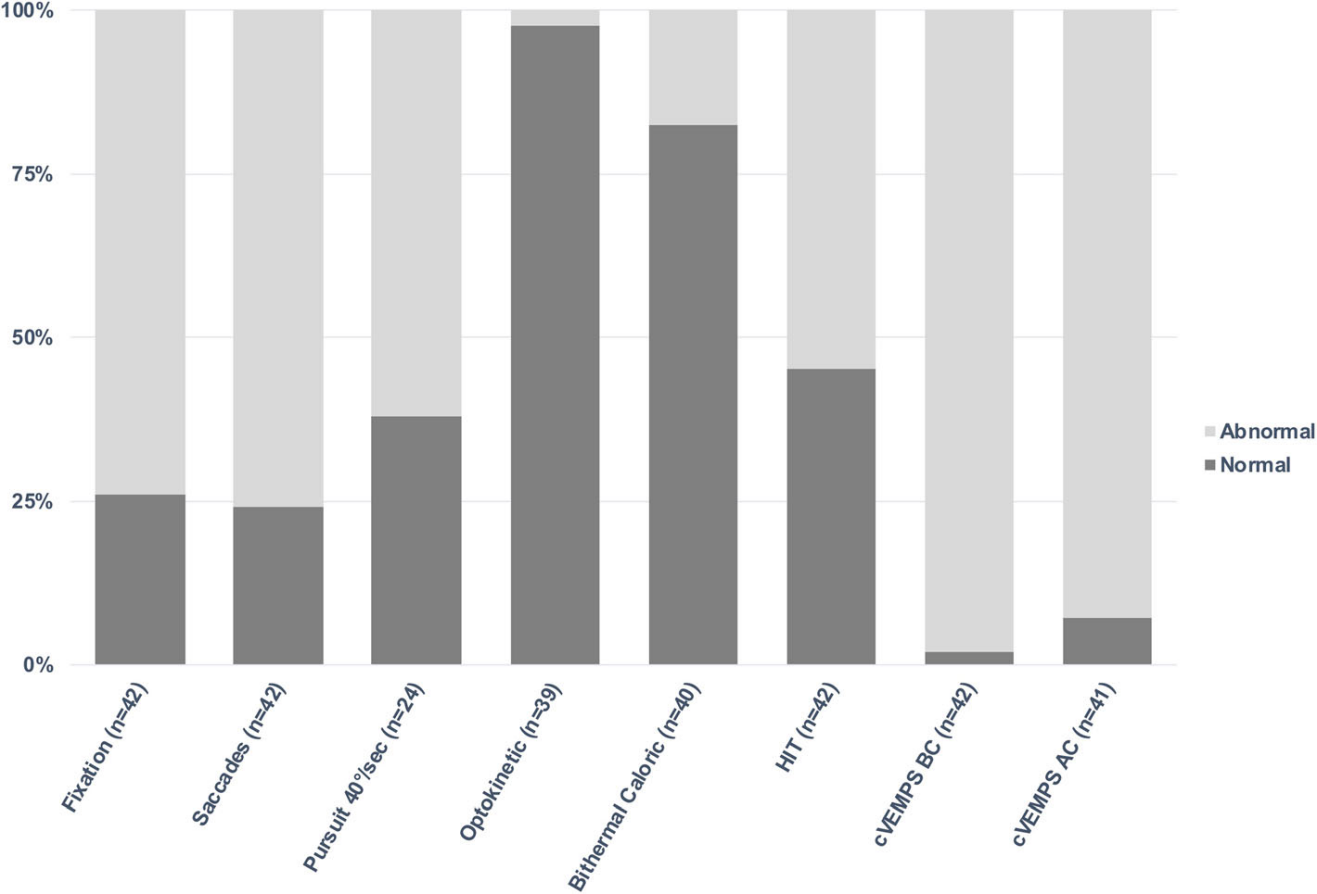
Proportion of subjects showing normal and abnormal oculomotor and vestibular tests

Canal function at low head rotation velocity was relatively well preserved. Normal symmetric bithermal caloric test was found in 82.5% (33/40) (Fig. 1). One subject had a complete absence of caloric response on both sides, two had bilateral hyporeflexia and four had asymmetrical responses. However, canal function at high head velocities (tested with clinical HIT) was impaired in 54.8% of our subjects (23/42) (Fig. 1).

The canal VOR function at middle head velocities (EVAR test) was analyzed in 39 subjects. The mean time constant for the entire population was 6.66 s ± 2.21 (mean time constant for per-rotatory and post-rotatory in clockwise and counter-clockwise rotations). It is significantly lower than the time constant assessed in a control population of 13-year-old children (13.3 s ± 3.0) and adults (18.9 ± 3.0) (One sample t-test, *p* < 0.001, verification of normality using Shapiro test) [31]. In patients with abnormal bithermal caloric test, the mean time constant is significantly shorter (4.19 s ± 1.14 with a range of 2.75–5.73 s) than in patients with normal bithermal caloric test (7.29 s ± 2.04 with a range of 3.76–11.56 s) (U Mann-Whitney test, *p* < 0.01). There was no significant difference between the time constant measured for per-rotatory responses and post-rotatory responses in the entire population.

The mean initial peak slow phase velocity (IPSPV) for the patient group was 20.85°/sec ± 6.14 (gain of 0.52). Since the value of the IPSPV is dependent of age [31], we compared the IPSPV obtained for the younger patients group (< 16 y.o.) and the older patients group (> 16 y.o.). Our results show that IPSPV in young patients (23.34°/sec ± 6.4) was significantly lower than the IPSPV found in a control group of 13-year-old children (46.7°/sec ± 9.0) (One sample t-test, *p* < 0.001, verification of normality using Shapiro test) [31]. The IPSPV was also significantly lower in adult subjects (> 16 y.o.) than in a control adult population (19.3 °/sec ± 5.54 vs 26.6°/sec ± 8.0, one sample t-test, p < 0.001, verification of normality using Shapiro test) [31].

Otolithic function was severely affected in our population. Only 7% (3/41) had normal c-VEMP with air conducted stimuli and only 2% (1/42) had normal c-VEMP with bone conducted stimuli (Fig. 1). Most subjects had absent c-VEMPs in both ears (49% in air conducted stimuli and 57% in bone conducted stimuli). The remaining ones had partial responses. There was no influence of age on the presence or absence of c-VEMPs response. Although insufficient muscle contraction could impair the VEMPS recording, this was not the case in our population since only responses obtained with an EMG above 100 μvolt were recorded.

### Hearing evaluation

**Fig. 2:**
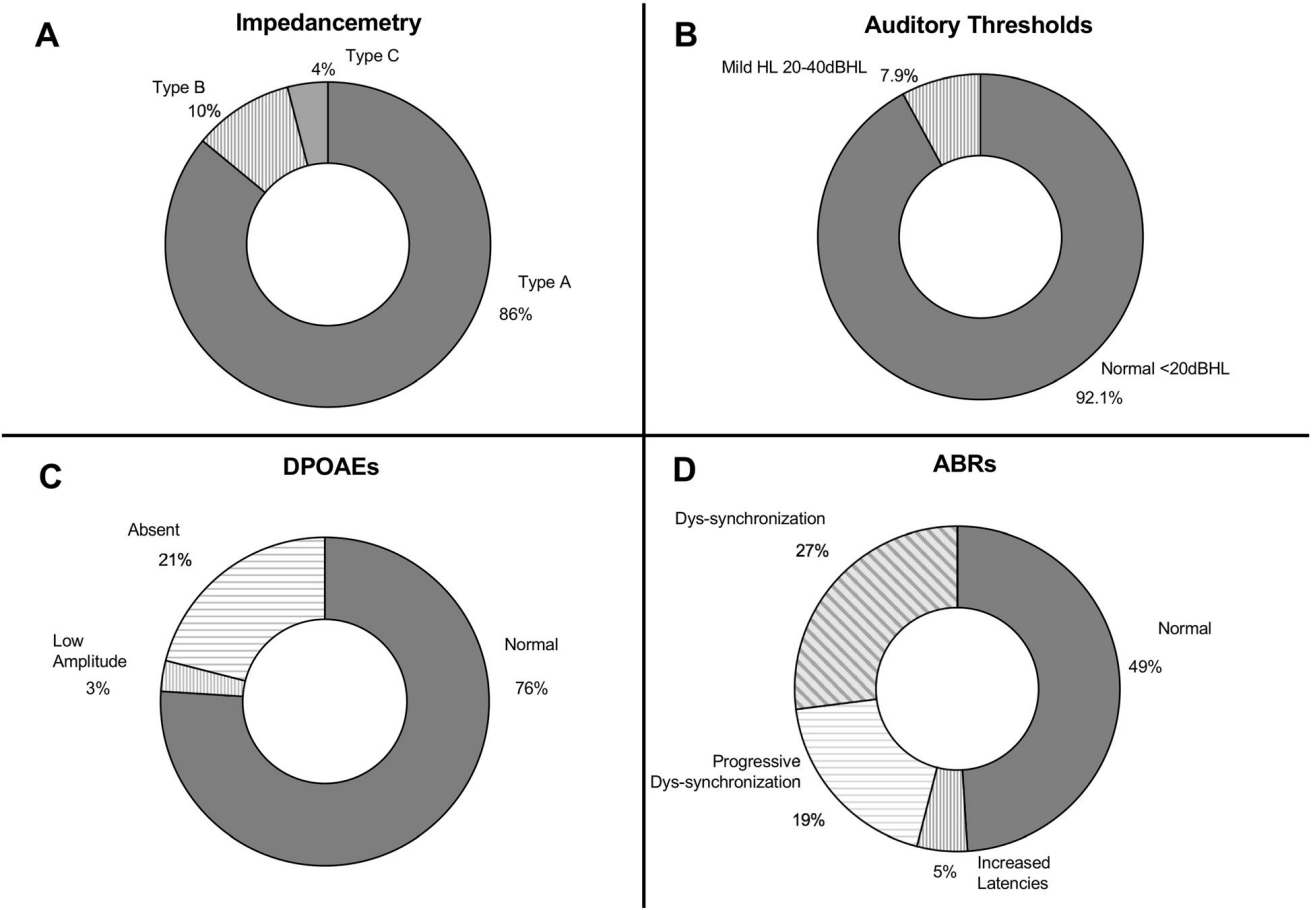
Hearing evaluation. Graphic representation of the prevalence of the various observations. Impedancemetry was performed on 41 subjects (82 ears); Hearing evaluation was performed on 40 subjects (80 ears); DPOAEs were evaluated on ears with normal type A tympanometry (69 ears); ABRs were performed on 39 subjects (78 ears)

**Fig. 3:**
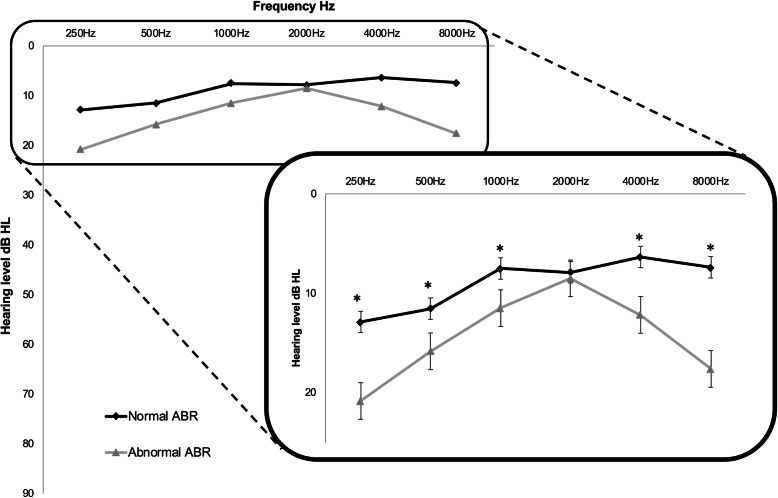
Hearing threshold of subjects with normal ABRs in black, and abnormal ABRs in grey. Auditory thresholds are slightly higher in low and high frequencies in the group of patients with abnormal ABR. (*) indicates frequencies with statistically significant difference (U Mann-Whitney test, *p* < 0,05)

Impedancemetry was done in 41 subjects (82 ears). Eighty-six percent of the tested ears had a normal type A curve (*n* = 71). Type B and type C curves were respectively observed in 10 and 4% of the tested ears (Fig. 2a). Hearing loss (based on audiometry) was diagnosed in 3 of the 40 tested children (7.5%) (Fig. 2b). All three children had mild bilateral SNHL. This SNHL was associated to normal tympanometry and abnormal ABRs. Out of the 71 ears with normal tympanometry, DPOAEs were performed in 69. They were normal in 76%, of low amplitude in 3% and absent in 21% of the tested ears (Fig. 2c). ABRs were recorded in 39 subjects (78 ears). Nineteen children had normal ABRs on both ears (49%). The others had either increased ABRs latencies (5%) with no sign of middle ear problem, or progressive dys-synchronization of the ABRs curves during the test (19%), or an immediate dys-synchronisation (27%) (Fig. 2d). Patients with abnormal ABRs had, in average, slightly higher pure-tone auditory thresholds than patients with normal ABRs (U Mann-Whitney test, *p* < 0,05) (Fig. 3).

### Oculomotor assessment

Gaze instability was observed in 74 % of our subjects (Fig. 1). Squared wave jerks (SWJ) were found in 74% (31/42) and flutters in 33% (14/42). All subjects with flutters also had SWJ. Gaze evoked nystagmus was observed in 38% (15/39).

Dysmetric saccades were common in our population (Fig. 1). Clinical evaluation of the saccades found hypermetric saccades in 76% of the patients (32/42). Five of those with hypermetric saccades also had hypometric saccades (5/32).

Electro-oculography recording of horizontal saccades (*n* = 38) and vertical saccades (*n* = 28) were analyzed. Mean horizontal latency was 242.1 ms (range 56.9–391 ms, SD 79.9 ms) and the mean vertical latency was 229.5 ms (range 71–473 ms, SD 84.3 ms). The analysis of saccadic accuracy demonstrated that only one subject was accurate to within 10% of the saccadic amplitude. Since the value of saccades latencies is dependent on age [36], we divided our population in a group of children younger than 16 years old (*n* = 18) and adults (over 16 years, *n* = 25). Our results show that the mean horizontal saccade latencies in our young patients (265.5 ms ± 63.4) and adult patients (230 ms ± 86) were not significantly longer than those observed in a control group of children (334 ms ± 26) and adults (211 ms ± 8) (One sample t-test, *p* < 0.001, verification of normality using Shapiro test) [36].

Pursuit was frequently impaired. At 10°/sec, pursuit was saccadic in 49% of our subjects (19/39). At 20°/sec it was saccadic in 56% (*n* = 22) and saccadic in 62% (*n* = 24) at 40°/sec (Fig. 1).

OKN was present in all tested subjects (39/39) and was asymmetrical in only one subject. All but one (38/39) could achieve a complete OKN/VOR inhibition by fixation of a close target during head rotation.

### Correlation between clinical parameters (time, severity and genetic factors) and audio-vestibular functions

**Fig. 4:**
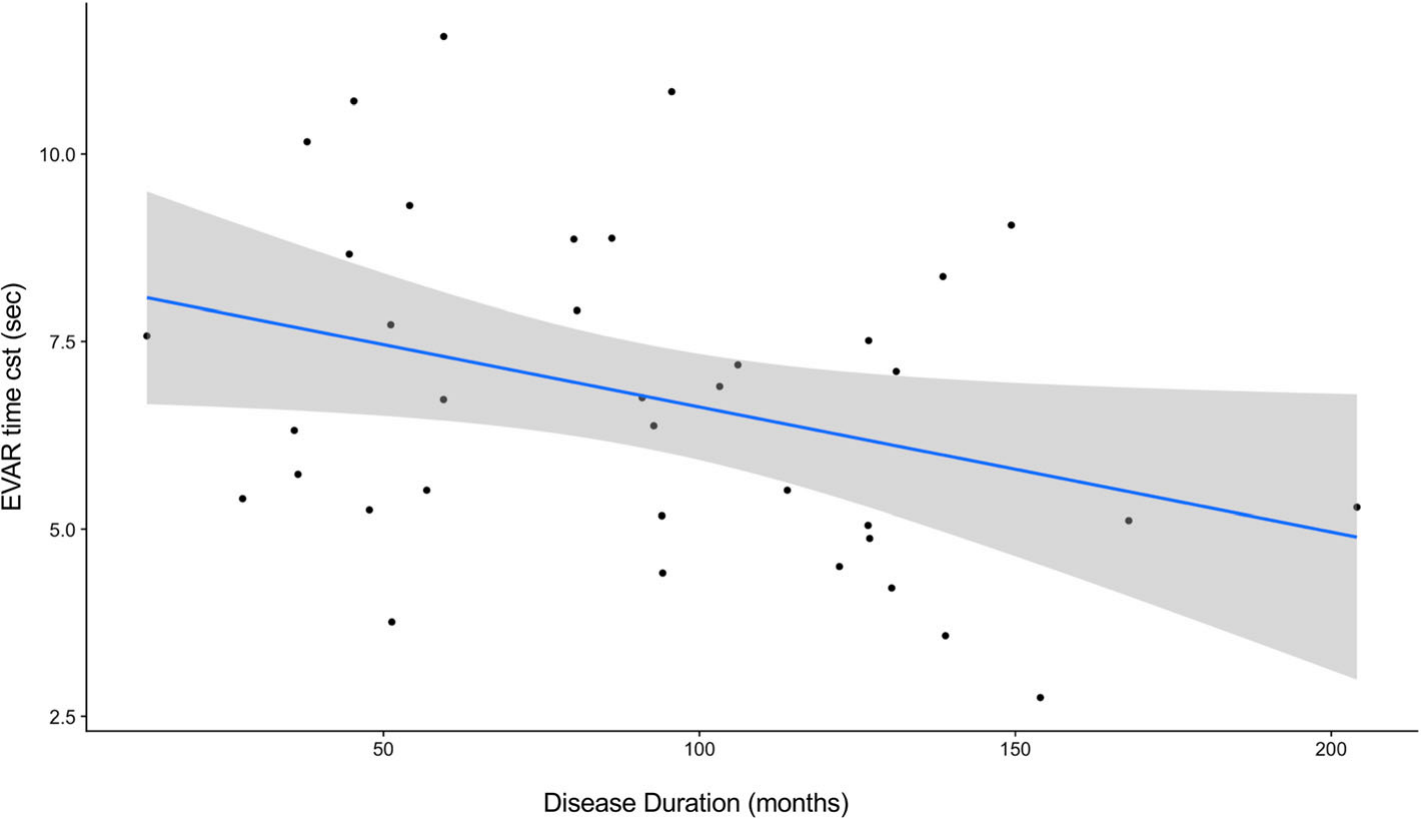
Correlation between disease duration and EVAR time constant. The longer the disease duration, the shorter the EVAR time constant (Pearson’s correlation, *r* = − 0.3419, *p* = 0.04)

Statistical correlation analysis shows that the time constant of EVAR responses (middle head velocities) decreased with the duration of the disease (Pearson’s correlation, *r* = − 0.3419, *p* = 0.04) (Fig. 4).

Abnormal ABRs, HIT and gaze instability (flutter, gaze nystagmus, square waves) are more frequently observed in patients with longer disease duration and in patients with higher FARS, ICARS and SARA scores (U Mann-Whitney test, *p* < 0.05, Table 2).

**Table 2:** Clinical characteristics in subjects with normal and abnormal auditory, vestibular and oculomotor measures. Statistical comparisons were done using U Mann-Whitney test. Median, Q25 and Q75 for all subgroups. Results in bold are significant

		Age at disease onset (years)		Age at visit (years)		Disease duration (months)		ICARS (total score)		FARS (total score)		SARA (total score)		Shorter GAA repeat length	
**ABR**	**Normal (*n*** **= 19/39)**	11.96 (7.26–13.64)	*P* = 0.13	16.39 (13.17–21.18)	*P* = 0.5	56.8 (39.6–119.0)	***P*** **= 0.004**	29 (19–39.5)	***P*** **= 0.0003**	57.75 (50.5–74.5)	***P*** **= 0.0001**	12 (9–16.38)	***P*** **= 0.0002**	700 (600–766)	***P*** **= 0.05**
	**Abnormal (*n*** **= 20/39)**	9.43 (5.94–10.29)		17.72 (15–20.69)		103.2 (81.6–126.9)		38,5 (33.5–45.3)		74 (68.6–81.5)		16.25 (13.5–18.63)		733 (600–875.25)	
**Caloric**	**Normal (*n*** **= 33/40)**	9.57 (6.16–13.43)	*P* = 0.644	16.41 (13.75–20.48)	*P* = 0.835	80.62 (51.24–114.12)	*P* = 0.147	32.0 (24–43)	*P* = 0.332	65 (55–76.5)	*P* = 0.182	13.5 (10.5–17.75)	*P* = 0.458	700 (600–833)	*P* = 0.172
	**Abnormal (*n*** **= 7/40)**	9.67 (7.92–10.30)		17.91 (12.7–21.06)		122.17 (99.12–136.81)		38.5 (30.5–44.25)		76.25 (70.25–83)		14.25 (11.88–21.88)		783 (766–867)	
**HIT**	**Normal (*n*** **= 19/42)**	10.06 (5.02–13.58)	*P* = 0.813	16.39 (12.03–18.51)	*P* = 0.159	70.97 (46.18–94.13)	***P*** **= 0.009**	26 (19–31.5)	***P*** **= 0.0004**	57.5 (48.63–70.38)	***P*** **= 0.005**	11 (9.5–13.25)	***P*** **= 0.0002**	733 (600–858)	*P* = 0.703
	**Abnormal (*n*** **= 23/42)**	9.76 (7.76–12)		17.53 (15.38–21.04)		114.12 (74.97–138.65)		42 (34.75–45.25)		76 (66.75–82.5)		16.75 (14.88–19.63)		700 (600–783.75)	
**Gaze Stability**	**Stable (*n*** **= 11/42)**	13.13 (6.39–14.52)	*P* = 0.177	15.5 (11.79–19.69)	*P* = 0.417	51.3 (44.58–80.62)	***P*** **= 0.007**	25.5 (18–35.75)	***P*** **= 0.026**	54.25 (43.37–54.25)	***P*** **= 0.014**	10.5 (9–15.63)	***P*** **= 0.014**	611.5 (540–733)	*P* = 0.163
	**Unstable (*n*** **= 31/42)**	9.42 (6.66–12.09)		16.82 (14.78–20.69)		98.7 (62.37–129.56)		35.5 (29.5–43)		70.75 (61.62–78.87)		15.25 (13–18.5)		733 (600–850)	
**Ocular Saccades**	**Normal (*n*** **= 10/42)**	12.38 (7.99–13.45)	*P* = 0.180	16.83 (13.17–20.62)	*P* = 0.873	56.84 (47.77–92.78)	*P* = 0.114	55 (48–59)	*P* = 0.142	26 (23–41)	***P*** **= 0.026**	11.5 (8.5–16)	*P* = 0.16	700 (600–800)	*P* = 0.536
	**Dysmetric (*n*** **= 32/42)**	9.76 (5.23–12.14)		16.62 (13.68–20.69)		94.13 (59.51–126.91)		70.25 (61.63–78.88)		35 (29–43)		14.75 (11.63–18.25)		716.5 (600–833)	

The presence of dysmetric saccades was associated with higher FARS scores (U Mann-Whitney test, *p* < 0.05, Table 2).

Subjects with abnormal ABRs tend to have longer triplet repeats (U Mann-Whitney test, *p* = 0.05, Table 2) but no correlation was found between the triplet repeats and the other oculomotor and vestibular parameters. For statistical analysis we used the number of triplets repeat on the shortest allele because it is considered as the most clinically relevant. Subjects with less than 800 triplet repetitions in the shortest allele are considered to have a less severe disease [37].

All data are presented in Table 2.

## Discussion

The aim of this study was to explore in detail the vestibular function in a large cohort of children and young adults with genetically confirmed FRDA, most of them being early onset patient (40/43), and to combine these observations to clinical, genetic, auditory and oculomotor evaluations. Our main findings are:

1. FRDA patients have vestibular dysfunction. Our patients’ canal function is abnormal at high and middle head velocities (assessed by HIT and EVAR) but normal for low head velocities (assessed by bithermal caloric test). Otolith function (assessed by cVEMP with bone and air conducted stimuli) is severely impaired.
2. FRDA patients have abnormal neural conduction in the central auditory pathways: 46% of our FRDA patients have ABRs dys-synchronisation.
3. Our FRDA patients present oculomotor abnormalities similar to those observed in previously published series [10, 12, 38]: fixation instability, saccades dysmetria, and saccadic pursuit.
4. These audio-vestibular and oculomotor anomalies are more frequently observed in subjects with longer disease duration and more severe forms of the disease (FARS, ICARS and SARA scores).

### Vestibular function

Previous studies have shown that vestibular anomalies are common to most FRDA patients [10, 11] but little is known concerning the peripheral or central origin of this deficit. We hereby discuss our vestibular results and see how these observations can help localize the neurophysiological impairment.

Our study shows that majority of our FRDA patients present partial vestibular impairment of canal function for high (HIT) and middle head velocities (EVAR) with mostly normal low head velocities canal function (bithermal caloric test). They also have severe impairment of otolith vestibulospinal function.

The time constant of EVAR responses is greatly reduced and this is linked to longer disease duration in our population. This observation corroborates previous findings of bilateral VOR deficit in FRDA patients [38]. Time constant of EVAR response is closely linked to the concept of “velocity storage”. This concept was introduced in 1979 by Raphan et al. to account for the fact that the VOR response to a rotatory impulsion followed by a constant velocity rotation lasts longer than the actual initial peripheral vestibular stimulation [39]. In normal subjects, the time constant of the observed nystagmus is two or three times longer than that of the corresponding activity in the vestibular nerve. The velocity storage concept suggests the presence of a neural integrator in the central vestibular system that extends the VOR [29]. This function is implemented in the cross commissural pathways between the right and left sided vestibular nuclei and under control of the cerebellum (e.g. the nodulus and uvula). Shorter time constant can therefore be linked to alteration of those central structures. But peripheral vestibular lesions can also lead to shorter time constant [34] and this hypothesis can be supported in our population by the observation of a shorter time constant in subjects with abnormal response to bithermal caloric test compared to those with normal caloric responses.

The prevalence of abnormal VOR at high head velocities might be under evaluated in our study since clinical HIT only detects canal impairment with at least 75% of canal functional loss. Even though the possible under evaluation, abnormal clinical HIT with clearly visible catch-up saccades is observed in more than half of our population. This test evaluates the reflex pathway from the vestibular canal receptors to the oculomotor nuclei. It is thought to be a purely peripheral vestibular test because it is too fast for other oculomotor control systems to participate (such as smooth pursuit, optokinetic, cervico-ocular reflex) [40]. However, abnormal clinical HIT has also been reported in pontine cerebellar stroke involving vestibulo ocular pathways or cerebellar lesion of the nodulus [41]. One of the limitations of clinical HIT is that, in FRDA, it can be difficult to differentiate true positive clinical HIT (where catch up saccade comes from an impaired canal) and false positive HIT (coming from the overlap of a hypermetric saccade or a saccadic pursuit with the VOR). Especially since the majority of our patients had abnormal saccades (either hyper or hypometric), abnormal eyes movements and saccadic pursuit. Video recorded HIT (vHIT) could differentiate false positive HIT (of central origin) from true positive HIT (of peripheral origin) and detect partial canal functional loss (less than 75%). This test was not available in our department at the time of the study.

To incriminate the cerebellar dysfunction for the anomalies observed in the VOR is tempting. But all of our subjects (except one) could perfectly inhibits VOR and OKN with fixation, and this observation does not support the hypothesis of a cerebellar impairment. Neurons important in VOR-suppression are located in the cerebellar flocculus and paraflocculus [42]. The ability to perform a VOR-suppression by fixation clearly differentiates FRDA from other cerebellar ataxia [43].

Two previous publications mention a higher prevalence of bilateral hyporeflexia in FRDA population with reduced caloric responses respectively observed in 66% (8/12) and 25% (4/16) of adults with FRDA [12, 38]. Unlike these observations, only two of our subjects (2/40, 5%) showed bilateral hyporeflexia and one a complete bilateral areflexia (1/40, 2,5%). The short duration of the disease in our young population might explain the low prevalence of caloric dysfunction, and this prevalence could increase as the disease evolves. A normal caloric function does not exclude peripherical vestibular impairment neither does it mean that the vestibular signs observed are strictly central. Indeed, preserved low frequency canal function can be observed in some peripherical inner ear diseases with selective canal impairment (reduced middle and high frequency canal function with normal low frequency canal function - e.i. Usher type I syndrome) [44].

Responses to otolithic stimulation are abnormal in almost all subjects with absent or high threshold cVEMPs responses. However, when a response was observed, latencies were normal in all but one subject. Although cervical VEMPs have mostly been applied to peripheral vestibular disorders, many studies report abnormal c-VEMPS response in various central pathologies, usually associated with long latencies [41, 45, 46]. The existence of mostly normal latencies in our population is in favor of a peripherical vestibular impairment. However, we did not test the robustness of the cVEMP responses with longer stimulation duration. Observation of signs of fatigability of the cVEMP responses could be an argument for neuropathy at the level of the vestibulospinal pathway.

The peripheric or central origin of our observed vestibular dysfunctions cannot be clearly determined based on our results. The vestibular impairment limited to middle and high head velocities but respecting low head velocities responses as well as the histopathological findings of no hair cells lesions but gliosis in the vestibular nucleus [18] and abnormalities of the spiral ganglion [19] and vestibular nerve [47] in FRDA patients could support the possibility of a vestibular neuropathy with central extension to the vestibular nuclei. But an association to a possible alteration of the peripheral vestibular system is not excluded especially since normal latencies were found in most of our cVEMPs responses. In order to determine the origin of the vestibular lesion further studies should be done in this population focusing mainly on:

- cVEMPs and oVEMPs and the latencies of the observed responses in prolonged stimulation
- video-HIT in order to precise both the gain but also the latency of the VOR

### Hearing anomalies

Clinically apparent hearing loss is relatively uncommon in patients with FRDA [4, 14, 48, 49] and, if present, is generally mild. In previous studies, only 8 to 13% have demonstrated elevated hearing thresholds [4, 14, 15]. Thirty-seven of the 40 tested subjects (92.5%) had normal pure tone audiometric thresholds (mean thresholds < 20 dB). Three subjects only had a mild bilateral SNHL.

However, even if FRDA patients have a normal pure tone audiometry they frequently report speech perception problems [15, 17]. These difficulties can be linked to disorders in the central auditory pathways. Some studies on FRDA patients report speech difficulties associated with absent or distorted auditory brainstem responses while pre-neural response from the cochlea hairs cells are normal [19, 20]. In the literature, the incidence of abnormal ABR varies from 30 to 100% [15, 17]. In our series, 51% demonstrated abnormal ABR (either increased latencies or dys-synchronization). Most papers link this observation to the presence of an auditory neuropathy/dysynchrony [50]. Neuro histological studies in patients with FRDA support this hypothesis. One study shows damage to the cochlear nerve without damage to inner ear structures [47] and another shows spiral ganglion degeneration [19]. A recent paper suggests that the Friedreich’s auditory neuropathy is linked to reversible energetic failure explaining why FRDA patients can have normal ABRs in response to short duration testing but abnormal ABRs for prolonged series of stimulation [21]. In our population, we observed dys-synchronization of the ABR waves appearing for long series of stimulation in 19% of our tested ears. Information regarding the hearing evaluation were extracted from a descriptive database and detailed information about the ABR measurements such as waves latencies could not be retrieved. Further studies with precise stimulation protocol, such as the one used in [21], should be done in order to document more precisely the ABR fatiguability.

Ell et al. observed a significant decrease in hearing acuity in the low frequency range in the Friedreich population compared to normal controls [10] in absence of conductive problems. The same pattern is found in our population, particularly the ones with abnormal ABRs (Fig. 2). Audiograms with increased low-frequencies thresholds are typically found at the beginning of inner ear diseases such as Meniere’s disease, or in certain forms of hereditary hearing loss. But such reverse slope audiograms are also described in both adults and children with auditory neuropathy spectrum disorder (ANSD) [50, 51]. Several explanations have been given for these reverse slope audiogram in ANSD: impairment of auditory processing of temporal information (since the timing of auditory nerve discharge play a role, particularly, in the encoding of low spectral acoustic signals) or neuropathological processes affecting particularly the apical fibers encoding for low-frequency sounds because of their longer course outside of the cochlear nucleus and greater axonal diameter [50]. This reverse slope audiogram was more pronounced in our subjects with abnormal ABR and this additional observation support the hypothesis of ANSD in FRDA patients. Signs of auditory neuropathy were more frequently observed in subjects with longer disease duration and seem to be associated with increased ICARS, FARS and SARA scores. Therefore, hearing difficulties can be considered as a sign of disease severity and disease progression.

Our findings confirm that children and young adults with FRDA likely face communication and educational challenges due to auditory neuropathy. For these patients, auditory tasks that are more complex than pure tone detection (most notably speech perception especially in noisy environment) may be dysfunctional far beyond what is indicated by the audiogram. All patient with FRDA should benefit from an auditory assessment including at least a pure tone audiometry, DPOAEs, ABR and a speech in noise test. In case of hearing difficulties, the introduction of personal FM system and communication training (i.e. listening tactics, lip-reading cues) proved to be helpful [15, 17].

### Oculomotor findings

The oculomotor abnormalities we found in our FRDA patients include fixation instability, dysmetric saccades and saccadic pursuit. The type and prevalence of the ocular motor anomalies observed are very similar to previously published series [10, 12, 38]. As opposed to other published series [11], we did not observe an increase of the overall mean saccade latencies for our population. This discrepancy might be explained by the relatively young age of our population associated to shorter progression of the disease. We did, however, observed a great variability in saccade latencies (range 56.9 ms to 391 ms). Analysis of saccadic accuracy demonstrated that 76% of our subjects were hypermetric and 12% hypometric. As previously observed [11], hypermetric and hypometric saccades were frequently combined in the same individuals.

## Conclusions

Our systematic vestibular and auditory evaluation of young subjects with confirmed FRDA highlights the prevalence of vestibular and auditory abnormalities. Our findings confirm that children and young adults with FRDA likely face communication and educational challenge due to their auditory neuropathy. FRDA is also characterized by severe and widespread anomalies in the oculomotor and vestibular systems. When combined with ataxia, these anomalies can further impair their autonomy.

The association of frequent saccadic intrusions, abnormal ABRs, decreased VOR gain and normal VOR-suppression by fixation is specific of Freidreich ataxia. Adding auditory and vestibular explorations to the evaluation of ataxic patients could help clinicians in the differential diagnosis of progressive ataxia syndromes.

Auditory brainstem responses, vestibulo ocular responses and gaze stability control are correlated to the disease severity and disease progression and could be used as markers of severity and progression. Therefore, testing these functions should be considered as additional tools for monitoring the evolution of the disease. All patient with FRDA should benefit from a vestibular and auditory assessment including at least v-HIT, EVAR, c-VEMPS, pure tone audiometry, DPOAEs, ABR and a speech discrimination test.

## Data Availability

The data are not publicly available due to the fact that the audio-vestibular evaluations were performed as part of the ACTFRIE trial and result from this trial have not been made public yet. However, the data that support the findings of this study are available from the corresponding author, upon reasonable request.

## Abbreviations

ABR: Auditory brainstem response
ANSD: Auditory neuropathy spectrum disorder
c-VEMP: cervical vestibular evoked myogenic potential
DPOAEs: Distortion product otoacoustic emissions
EVAR: Earth vertical axis rotation
FRDA: Friedreich ataxia
FARS: Friedreich Ataxia Rating Scale
HIT: Head impulse test
ICARS: International Cooperative Ataxia Rating Scale
IPSPV: Initial peak slow phase velocity
OKN: Optokinetic nystagmus
o-VEMPS: ocular vestibular evoked myogenic potential
SARA: Scale for Assessment and Rating of Ataxia
SNHL: Sensorineural hearing loss
SWJ: Square wave jerks
v-HIT: video head impulse test
VOR: Vestibulo-ocular reflexes
